# Point Prevalence Studies Are Necessary as First Steps in Studying the Epidemiology of Viruses and Other Pathogens Transmitted by Arthropods

**DOI:** 10.3390/v14061244

**Published:** 2022-06-08

**Authors:** Charles H. Calisher

**Affiliations:** Arthropod-Borne and Infectious Diseases Laboratory, Department of Microbiology, Immunology and Pathology, College of Veterinary Medicine and Biomedical Sciences, Colorado State University, 3195 Rampart Rd., Delivery Code 1690, Foothills Campus, Fort Collins, CO 80523-1690, USA; calisher@cybersafe.net; Tel.: +1-(970)-227-9849

**Keywords:** longitudinal, long-term, surveillance, field studies, phleboviruses, sand flies

## Abstract

Studies of the epidemiology of arthropod-borne viruses are based on understanding whether a given virus is found in a particular location or locations, how prevalent the virus is in that area, which vertebrate hosts serve as reservoirs of the virus, the relationship between the size of the populations of these reservoirs and the prevalence of the virus and, of course, whether the virus causes human, livestock, or wildlife diseases, as well as other characteristics. However, seasonal conditions fluctuate, annual conditions fluctuate, human impacts alter the environment, and the habitat and ecosystems naturally change. Because these parameters provide a more encompassing view of the natural history of a virus, it is important to recognize that point prevalence studies comprise only a single view of the situation and that longitudinal studies are required to obtain a more complete and useful understanding of the natural history of the virus. This paper provides details as to whether and how to conduct such studies.

First, an illness is recognized, then perhaps diagnosed: three dead, 20 ill, of which eight have been hospitalized. These numbers comprise a point prevalence, the proportion of a population that analyzes the illness at a specific point in time. The numbers represent the numerators in the formula “numerator/denominator”, but what are the denominators? Such denominators are needed in order to determine the severity of an illness and its potential impact upon the extant population. If in this example the total population is four million (people, sheep, dogs, cattle, etc.) the medical problem is low, at least at first glance. If the total population is 23, i.e., 23/23, that is another story. Determining the cause of this illness and how it is transmitted and acquired, as well as its potential to spread to other populations, then is necessary in order to prevent the occurrence of additional cases. Recognizing the presence of a previously unknown pathogen, such as by polymerase chain reaction (PCR), is a critical first step in this process but, at this stage, definitively tells us only a minimum regarding the means by which the pathogen is transmitted, where and when it arose, how it persists, or what its potential is to spread to other populations. Such determinations depend on epidemiologic studies.

The first step in understanding the epidemiology of arthropod-borne viruses, including those that cause sand fly fevers, and other potential pathogens, is to determine whether that pathogen is found in a given location. Usually, such determinations are made in the area where a patient or patients have been identified. After that, geographic areas where such a pathogen is suspected to be endemic are investigated. Sand fly-borne phleboviruses are no exception to this approach and efforts are made to collect sand flies (Phylum: Arthropoda, Class: Insecta, Order: Diptera, family Psychodidae, subfamily Phlebotominae).

The name sandfly (or sand fly) caused misunderstandings, because these insects are not always related to sandy environments. Moreover, in some parts of the world, the name sand fly is applied to other dipterans, such as black flies (Simuliidae) and biting midges (Ceratopogonidae) [1]. Some suggest that this name is derived from the color of the insects, rather than to their habitat [1]. The taxonomy of sand flies is still debated; a conservative and widely used approach recognizes six genera in the subfamily Phlebotominae, three from the Old World (Phlebotomus, Sergentomyia, Chinius) and three from the New World (Lutzomyia, Brumptomyia, Warileya) [2].

Sand flies are collected using any of a variety of methods, such as sticky traps, which use castor oil and immobilize insects, but recovery from which live specimens are difficult; CDC miniature light traps [3]; and many other devices. Sand flies also are commonly captured manually using aspirators. Regardless of the method of capture, it is useful to maintain the captured insects as live samples, so that the pathogens they might contain remain viable for studies other than insect identifications, for specific pathogen identification, or to search for hitherto unrecognized viruses or other pathogens in new locations.

Distribution and abundance assessments must be made, and for these, longitudinal designs are recommended for the collection of data on population dynamics. Longitudinal surveys are research studies that involve repeated sampling of the same sites over time [4]. A limited number of sites (as determined by local capacity) are selected by predefined geographical range, biotope type/s and time frame are monitored over one or more (preferable) vector seasons. These surveys can be used to assess vector presence or absence and to determine their population dynamics (e.g., seasonality, density peaks, number of generations) and abundance. Density peak assessments are required to illustrate time windows for abundance sampling. If combined, both types of data can later be used for disease risk assessment. Data on presence or absence can be collected by a number of appropriate sampling methods but the estimation of population dynamics and population abundance should be carried out by a single sampling method in accordance with a standardized protocol or using consistently applied multiple sampling protocols. Note that not every species responds in the same way to each trap type. For example, *Ph. perfiliewi* and *Ph. perniciosus* have different phototropisms.

To understand the dynamics of a given phlebovirus or other potential pathogen, long-term studies of the target vector and pathogen in question are needed. That is because normal (and certainly abnormal) fluctuations occur regarding temperature, precipitation, and land use, productivity levels of food sources for endemic vertebrates, introduction of members of non-native species, changes in species diversity at the site, the occurrence of forest fires, landslides, floods, and volcanic eruptions, greenhouse gas levels, local carrying capacity of the site and many more complicating factors. When one or more of these interdependent features vary, the effect can be significant. For example, drought can cause a reduction in plant productivity or a decrease in productivity of plants of one species and increase in productivity of another species. Elevated or decreased temperature changes can cause relevant changes in plant or insect productivity or even presence. Thus, food sources can change to a point at which they effect the populations of contemporary dependent populations.

Usually, many so-called long-term studies of habitats are conducted over rather short terms, i.e., days or weeks. However, in order to genuinely understand what is occurring at a particular site, monitoring that site for multiple months or even years is required. Funding for such studies is difficult to obtain because observable changes may not occur until an event, or series of events, happens. For example, a particular plant may serve as a resource for resident vertebrates on which insects might feed and when that plant population is reduced those insect populations may be concomitantly reduced. Likewise, an increase in productivity of that plant may lead to a considerable increase in the population of that insect.

Because ecosystems are usually quite stable, changes in them do not occur weekly or monthly, perhaps even not annually. Genuine long-term studies become seemingly tedious, expensive, and wasteful of labor but without them all that can be acquired is a series of point prevalences useful for gathering numerators. A scant few, if any, true longitudinal studies of phleboviruses in nature have been done. Many such studies regarding leishmanias in nature have been published, as exemplified by references [5,6,7]. Results from true longitudinal studies can be correlated with human infections occurring during the same periods and provide information useful for preventing disease transmission.
